# Prevalence of vitamin B 12 deficiency and associated factors among primary school children: North East Ethiopia: multicenter cross-sectional study

**DOI:** 10.1186/s41043-024-00568-6

**Published:** 2024-06-12

**Authors:** Ermiyas Endewunet Melaku, Besufekad Mulugeta Urgie, Alemnesh Tesema Tilahun, Hilina Ketema Assefa, Alemayehu Abera Abebe, Aklile Semu Tefera

**Affiliations:** 1 School of Medicine, Debre Berhan University, Debre Berhan, Ethiopia; 2 Department of Laboratory, Debre Berhan University, Debre Berhan, Ethiopia; 3 Department of Nursing, Debre Berhan University, Debre Berhan, Ethiopia; 4 Department of Horticulture, Debre Berhan University, Debre Berhan, Ethiopia; 5 Department of Epidemiology, Debre Berhan University, Debre Berhan, Ethiopia

**Keywords:** Vitamin B12 Deficiency, Primary school students, Ethiopia

## Abstract

**Background:**

The prevalence of Vitamin B12 deficiency is common and is more frequent in low- and middle-income countries with a poor or inadequate diet of animal foods. In Ethiopia, researches related to the status of micronutrients in children are limited. Therefore, this study aimed to assess the prevalence of vitamin B12 deficiency and associated factors among primary school children.

**Methods:**

A cross-sectional study design was conducted from January 10-February 30/2023. A total of 514 students were selected using a systematic random sampling technique. Face-to-face interviews using a structured questionnaire, document review, anthropometric measurement, and laboratory studies were implemented to collect data. Data was analyzed by STATA version 14 and summarized by using frequency tables and graphs. Logistic regression analysis was done to identify factors associated with vitamin B12 Deficiency.

**Results:**

About 34% of the students were found to have vitamin B12 deficiency. Not Consuming animal products (AOR = 1.83, 95% CI:1.20–2.79) and low body mass index (AOR = 1.62, 95% CI:1.05–2.47) were associated with vitamin B12 deficiency.

**Conclusions:**

The study revealed a notable high deficiency of vitamin B12 in primary school students. Consumption of animal products and BMI were identified as statically significant associated factors with serum concentration of vitamin B12.

## Introduction

Vitamin B12, also known as cobalamin, is a water-soluble vitamin that serves as a coenzyme. It plays a crucial role in promoting a healthy neural system and the formation of red blood cells, making it indispensable during early growth stages [1]. Like many other organisms, humans fulfill their nutritional needs for vitamin B12 through a combination of gut microbial biosynthesis and dietary intake [2].

Deficiency of vitamin B12 is considered to be a global health burden as a result of increasing evidence for its role in neural tube development, growth, immunity, and cognitive functioning [3]. Vitamin B12 deficiency in children is a significant preventable public health problem with potential long-term neurological consequences, if left undiagnosed. It is often underreported in children from developing countries, with a varying prevalence of 21–45% [4]. Vitamin B12 deficiency is often underreported in children from developing countries, with a varying prevalence of 21–45% [5].

The prevalence of Vitamin B12 deficiency is common and is more frequent in low- and middle-income countries with a poor or inadequate diet of animal foods. In the United States and the United Kingdom, the prevalence of vitamin B12 deficiency is approximately 6% in adolescents. Latin American countries have a clinical or subclinical deficiency rate of approximately 40%. The prevalence was 70% in Kenyan school children, 32.4% in healthy Indian school-going adolescents, and 16.5% in Australian immigrants [3, 6–10].

A high prevalence of Vitamin B12 deficiency was found to be associated with poor intake of animal products, Poverty, age, sex, place of residency, maternal education, and malnutrition [3, 8, 11–14] B12 deficiency can affect individuals of all ages. However, infants, children, adolescents, and women of reproductive age are at high risk of deficiency in populations where dietary intake of B12-containing animal-derived foods is restricted [15].

In Ethiopia, researches related to the status of vitamin B12 in children are limited. A single cross-sectional study conducted on school aged children about 14 years back showed more than two thirds of the school-aged children (79.5%) had at least one micronutrient and 40.5% had two or more coexisting micronutrient deficiencies [16]. Therefore, this study was aimed at showing the prevalence of vitamin B12 deficiency and factors associated with it.

## Methods and materials

### Study subjects and settings

A cross-sectional study was conducted from January 10 to February 30, 2023, at four randomly selected primary schools in the North Shoa district of Ethiopia. The study was carried out at Keyit, Hagere Mariam, Deneba, and Tarma Ber primary schools. The study subjects were first-cycle primary school students (grades 1–4) attending at these selected schools during the study period. The number of first-cycle primary school students during the study period was 350, 375, 405, and 430 at Keyit, Hagere Mariam, Deneba, and Tarma Ber primary schools, respectively. Accordingly, 121, 130, 140, and 148 students were selected from Keyit, Hagere Mariam, Deneba, and Tarma Ber primary schools, respectively based on the proportion of students each school and class had at the time of data collection. Students who had been free from any acute infection in the past month were included in the study.

### Sample size and sampling procedure

The sample size was calculated using a formula for estimation of single population proportion with the assumption of 95% confidence interval, 5% margin of error, and prevalence of 32.4%. Systematic Random sampling method was used to recruit 539 sampled students [17].

### Socio-economic data

Data were collected through trained data collectors (nurses and public health officers) using a predesigned semi-structured questionnaire. Students and parents/guardians were interviewed to obtain sociodemographic and socioeconomic data.

### Anthropometric measurements

Focused clinical examination was done for each of the students. A digital weighing scale with 250.00 kg capacity (Seca787) was used to obtain the weights of the students. A non-distensible plastic tape with 200.00 cm capacity was used to measure the heights of students with the child leaning against a wall and looking straight ahead, ensuring that the legs are straight and touching together, arms are at the sides, and shoulders are level, to ensure uniformity.

### Dietary assessment

A 24-hour dietary recall checklist of 12 food groups proposed by Food and Agricultural Organization [18] was used to elicit dietary diversity scores of the students. Dietary diversity scores (DDS) were calculated by summing up the points scored in each of the food groups and classified as low (≤ 4), medium [5–8], and high [9–12].

### Blood sampling and biochemical analysis

About 5 ml of whole blood sample was collected from each student using Serum-Separator tube at the school level and transported at 2–8◦C. The samples were stored at -20◦C for one month before analysis was made. The stored samples were then transported to Ethiopian Public health Institute laboratory for biochemical analyses. Serum vitamin B12 concentrations was determined using Electrochemiluminescence immunoassay method by Cobas e404 Analyzer machine.

### Data analysis

Data were coded, recoded, cleaned, and explored to identify missing values and inconsistencies. Data were entered into EpiData V.3.1 and analysed by STATA 14.0. In the descriptive analysis, the mean with SD, frequency, and percentages were calculated. Basic assumptions of logistic regression were checked. Both bivariate and multivariate logistic regression analyses were used to identify factors independently associated with vitamin B12 concentration of students. Adjusted odds ratio (AOR) with 95% confidence interval (CI) and *p* value < 0.05 were used to identify factors associated with serum concentration of vitamin B12 of students.

### Definition of terms

- Vitamin B 12 Deficiency: Is defined as a serum vitamin B12 concentration of less than 150 pg/ml.
- Dietary diversity scores (DDS): calculated by summing up the points scored in each of the food groups and classified as low (≤ 4), medium [5–8], and high [9–12].

## Results

### Sociodemographic characteristics of children and parents

A total of 514 primary school students were included in the study. About 55% of the students were males and the mean age of the students was 10.5 ± 0.28 years. About 54% of the students were from urban areas. Approximately 75% of fathers and 67% of mothers have completed at least primary school. About 46% of mothers and 44% of fathers were farmers. The average number of family sizes was 5.67 ± 0.07. (Table 1).

**Table 1:** Sociodemographic characteristics of the study participants at selected first cycle primary schools of North Shoa district, North East Ethiopia from January 10-February 30/2023 (*N* = 514)

Variables (*N* = 514)	category	Frequency (*N*)	Percent (%)
Sex of Students	Male	285	55.45
	Female	229	44.55
Residency	Urban	276	53.7
	Rural	238	46.3
Mothers’ education	Cannot read and write	78	15.17
	Read and write only	109	21.20
	primary school completed	174	33.85
	Secondary school completed	78	15.17
	College and above	75	14.59
Fathers’ Education	cannot read and write	45	8.75
	Read and write only	91	17.70
	Primary school completed	186	36.12
	Secondary completed	85	16.54
	College and above	107	20.82
Mothers Occupation	Farmer	231	46.02
	Government employee	182	36.25
	Merchant	62	12.35
	Others	27	5.42
Fathers’ Occupation	Farmer	211	44.14
	Government employee	145	30.33
	Merchant	86	17.99
	Others	36	7.53
Religion	Orthodox	447	87.05
	Muslim	44	8.5
	Protestant	15	3.0
	Other	8	1.5
Family income in ETB	< 2500	251	49.70
	2500–5000	189	37.43
	5000 and above	74	12.87

### Individual dietary diversity score of the students

**Fig. 1:**
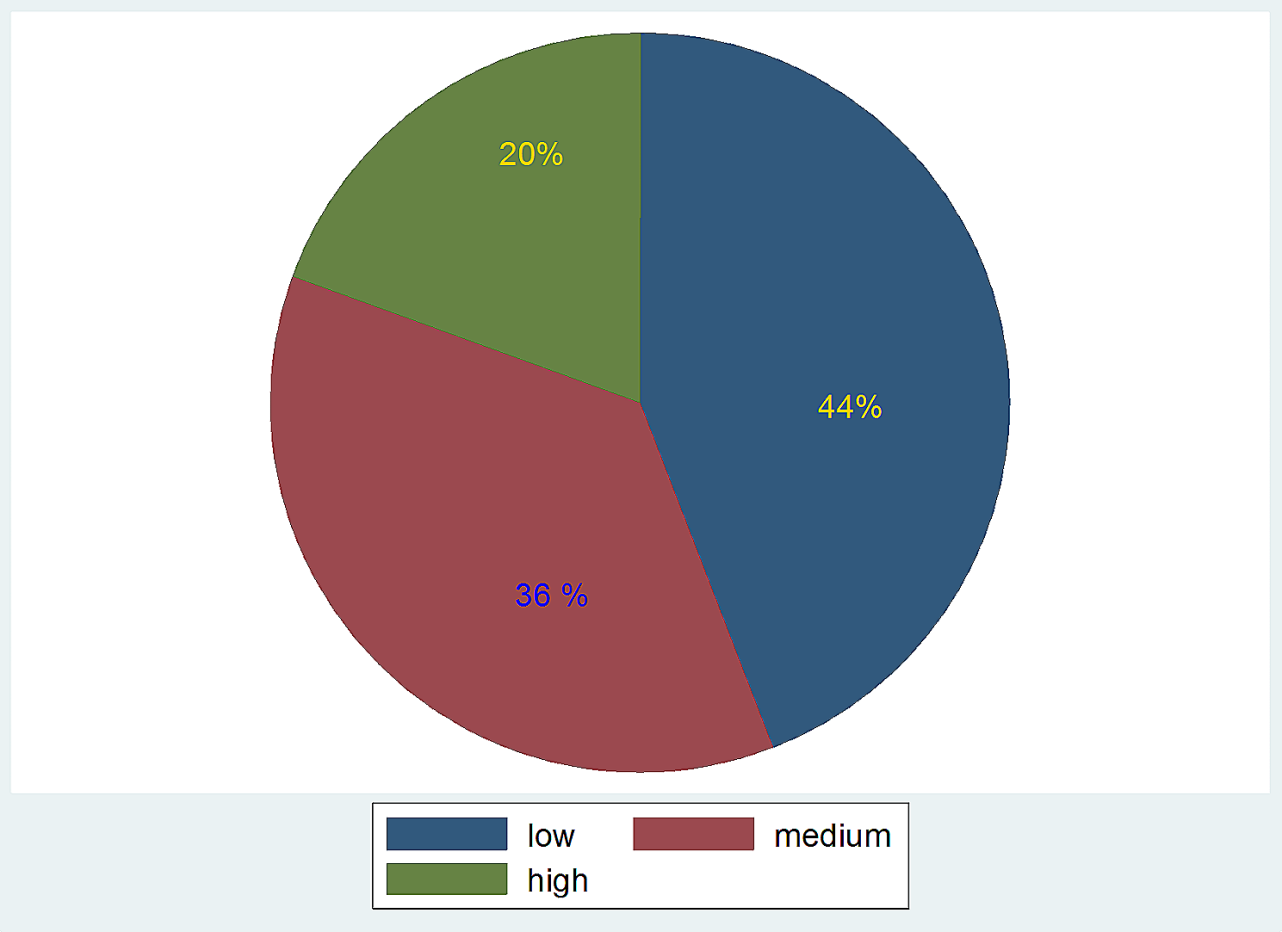
24-hour dietary diversity score of students at selected primary schools of North shoa Zone, North East Ethiopia from January 10-February 30/2023 (*N* = 514)

When the 24-hour individual dietary diversity status was assessed, about 44% (227) students had low dietary diversity score (≤ 4 food groups) and 36% (187) of students had medium dietary diversity score (5–8 food groups). Only 20% of students were found to have high dietary diversity score (9–12 food groups (Fig. 1).

### Nutritional assessment

The prevalence of stunting among students was 34.5%. The mean body mass index of students was 15.75 ± 0.072. About 32.5% of students had low Body mass index (underweight) and 61.5% of students were found to have normal Body mass index. The rest (6%) of students were overweight. About 52% of stunted and 56.4% of underweight students were females.

### Vitamin B 12 containing food consumption of the students

Of the total 514 students, about 34.43% (177) students had consumed at least one of the animal products (meat and meat products, milk and milk products, fish and chicken) in the preceding 24 h. Meat and milk products were the most frequent animal products consumed by students (Table 2).

**Table 2:** Vitamin B12 containing Food groups consumed by students at selected first cycle primary schools of North Shoa district, North East Ethiopia from January 10-February 30/2023 (*N* = 514)

Variables	Category	Frequency (*n*)	Percent (%)
Meat and Meat Products	Yes	98	19.06
	No	416	80.94
Milk and milk products	Yes	156	30.35
	No	358	69.65
Egg	Yes	142	27.63
	No	372	72.39
Fish	Yes	8	1.57
	No	502	98.43
Chicken	Yes	15	2.92
	No	499	97.08

### Vitamin B12 Deficiency

The mean serum vitamin B12 level of students was 229.6 ± 6.52(95% CI:216.7732-242.4017). About 34% (176) of students had vitamin B12 deficiency (serum B12 level < 150 pg/ml). Lower proportion of students with age less than 10 (38%) had low vitamin B12 level compared to students with age ten and above (48%). About 32% (73) of female and 36% (103) of male students in in this study had vitamin B12 deficiency.

Bi-variate and multi-variate logistic regression analyses were used to identify associated factors for vitamin B12 deficiency. Accordingly, mothers’ education level, mothers’ occupation, residency of the children, family size, eating animal products, dietary diversity score, and BMI of students were found to have *p* values of less than 0.2. Variables with *p* values less than 0.2 in the binary logistic regression were entered into the multivariate logistic regression. After computing the multiple logistic regression intake of animal products and BMI of students were found to have a statistically significant association with serum vitamin B12 level at *P* value of less than 0.05. Age and sex of students were not found to have statically significant association with vitamin B12 deficiency (Table 3).

**Table 3:** Binary and multiple logistic regression analysis of variables associated with vitamin B12 deficiency at selected first cycle primary schools of North Shoa district, North East Ethiopia from January 10-February 30/2023 (*N* = 514)

Variables	Category	Vitamin B12		Crude OR with 95% CI	Adjusted OR with 95%) CI	*P* value
		Normal	Low			
Mothers Education	Secondary and above	109	40	1.61 (1.06–2.46)	1.09 (0.84–1.08)	0.469
	Below secondary	228	135			
Mothers’ occupation	Government employer	146	152	1.78 (1.22–2.60)	1.15 (0.55–2.39)	0.705
	Farmer	63	96			
Family size	Treated as a continuous variable			0.91 (0.81–1.02)	0.95 (0.83–1.08)	0.469
Residency	Urban	169	69	1.55 (1.07–2.24)	1.62 (0.80–3.26)	0.271
	Rural	169	107			
Animal product intake	Yes	126	51	1.45 (0.98–2.16)	1.83 (1.20–2.79)	0.005*
	No	212	125			
Dietary Diversity Score	Low	171	56	4.77 (3.95–9.13)	1.76 (0.53–1.12)	0.167
	Medium/high	112	175			
BMI of children	Normal	250	112	1.62 (1.09–2.40)	1.62 (1.06–2.47)	0.027*
	Underweight	88	64			

Abbreviation: BMI = Body Mass Index, CI = Confidence Interval, OR = Odds Ratio

*= Statically Significant

## Discussion

This study was aimed to assess the prevalence of vitamin B12 deficiency and associated factors at selected first cycle primary school students of North Shoa district of North East Ethiopia. The findings revealed that approximately 34% of students had low serum vitamin B12 concentration. While deficiencies of vitamin B12 are rare in Western countries, they are more prevalent in developing and low-income countries [19].

A Study conducted in Nepal and India also showed similar findings of vitamin B12 deficiency [3, 9]. Compared to the studies conducted in Mexico, Colombia and Israel, this study showed higher prevalence of vitamin B12 deficiency [9, 12, 20]. On the other hand, studies conducted in Latin America, school aged children in Kenya, and Bhutan revealed higher prevalence of vitamin B12 deficiency compared to this study [21–23].

In Ethiopia, most research on vitamin B12 status has focused exclusively on pregnant women. While this limits our ability to directly compare our results with similar age groups, we still find these data valuable as they serve as a foundational point for further exploration of this topic. Expanding research beyond pregnant women to include other demographic groups, such as children or non-pregnant adults, could provide a more comprehensive understanding of the prevalence and impact of vitamin B12 deficiency in the population. Therefore, we view our findings as a starting point for initiating broader investigations into this important health concern.

The consumption of animal products was found to be positively correlated with the serum concentration of vitamin B12 in a multiple logistic regression analysis. Students who consumed animal products within the previous 24 h had a serum vitamin B12 level that was approximately 1.8 times higher than those who did not consume animal products. (AOR = 1.83, 95 CI:1.20–2.79, *P* = 0.005). The positive correlation between the consumption of animal products and serum concentration of vitamin B12 can be attributed to the fact that animal-derived foods are the primary dietary sources of vitamin B12 for humans. Vitamin B12 is predominantly found in animal products such as meat, poultry, fish, eggs, and dairy products. Therefore, individuals who consume these foods regularly are more likely to have higher levels of vitamin B12.

Similar research conducted in Mexico also demonstrated a positive association between daily milk intake and maintaining normal serum concentrations of vitamin B12. This correlation suggests that individuals who consume milk regularly may have higher levels of vitamin B12 in their bloodstream [21]. Other studies conducted in India and Nepal have similarly reported associations between vitamin B12 deficiency and the consumption of animal products [9, 24].

Students with a normal BMI exhibited a serum vitamin B12 concentration approximately 1.6 times higher than that of students classified as underweight based on their BMI (AOR = 1.62, CI:1.06–2.47, *P* = 0.027). The observed positive association between vitamin B12 concentration and BMI could be explained by the possibility that Students with a normal BMI may have a more balanced and varied diet, including a sufficient intake of vitamin B12-rich foods such as meat, poultry, fish, eggs, and dairy products.Other comparable studies conducted in Turky, Netherlands and Germany have also indicated a positive correlation between BMI and serum vitamin B12 concentration [25–27].

### Clinical implication of the study

The study highlighted a substantial portion of students may be at risk of vitamin B12 deficiency in the country. Identifying these individuals could allow for targeted interventions to prevent or address vitamin B12 deficiency-related health problems.

Vitamin B12 deficiency can lead to various health consequences, including anaemia, neurological impairments, developmental delays in children, and compromised immune function. Recognizing low serum vitamin B12 levels in students prompts healthcare providers to assess for potential symptoms or signs of deficiency and initiate appropriate treatment or supplementation as needed.

Vitamin B12 plays a crucial role in cognitive function and brain development. Students with low serum vitamin B12 levels may experience difficulties in concentration, memory, and overall academic performance. Addressing vitamin B12 deficiency can potentially improve cognitive function and positively impact educational outcomes.

The findings also underscore the importance of routine nutritional screening particularly among children and adolescents. Healthcare providers can use this information to advocate for dietary diversity, encourage consumption of vitamin B12-rich foods, and promote awareness of the importance of adequate nutrient intake for overall health and well-being.

## Conclusions and recommendations

The findings of this study revealed a notable high deficiency of vitamin B12 in primary school students of the study area compared to the studies reported from other regions. Consumption of animal products and BMI were identified as statically significant associated factors with serum concentration of vitamin B12.Raising awareness of parents and guardians about the importance of a balanced diet, particularly on sources of vitamin B12 should be emphasized. Further researches to explore additional factors influencing vitamin B12 deficiency in primary school students has to be encouraged. This may include socio-economic factors, dietary preferences, disease-related factors and cultural influences that could provide a more comprehensive understanding of the issue.

### Strength and limitation of this study

#### Strengths

This is a multi-center study thereby enhancing the generalizability of results to broader populations. Laboratory determination of serum vitamin B12 levels may be important to study the subclinical vitamin B12 deficiency. Moreover, this study stands out as the pioneering laboratory-based investigation of vitamin B12 specifically targeting primary school students in the country, filling a critical gap in the existing literatures. By offering novel insights into vitamin B12 deficiency in children, the study contributes significantly to advancing our understanding of this important health concern.

#### Limitations

It is important to acknowledge that certain associated factors, such as disease related factors for vitamin B12 deficiency, may not have been investigated in this study. While the study may have focused on certain factors contributing to vitamin B12 deficiency among primary school students, it is possible that other variables, such as underlying health conditions or medical history, were not explored.

## Data Availability

The data used and/or analyzed during the current study are available from the corresponding author on reasonable request.
